# No Evidence for Biased Attention Towards Emotional Scenes in Bornean Orangutans (*Pongo pygmaeus*)

**DOI:** 10.1007/s42761-022-00158-x

**Published:** 2022-11-24

**Authors:** D. W. Laméris, E. van Berlo, T. S. Roth, M. E. Kret

**Affiliations:** 1 Behavioural Ecology and Ecophysiology Group, Department of Biology, University of Antwerp, Wilrijk, Antwerp, Belgium; 2 Antwerp ZOO Centre for Research & Conservation (CRC), Royal Zoological Society of Antwerp (RZSA), Antwerp, Belgium; 3 Cognitive Psychology Unit, Institute of Psychology, Leiden University, Leiden, The Netherlands; 4 Institute for Biodiversity and Ecosystem Dynamics, University of Amsterdam, Amsterdam, The Netherlands; 5 Amsterdam Brain and Cognition, University of Amsterdam, Amsterdam, The Netherlands; 6 Apenheul Primate Park, Apeldoorn, The Netherlands; 7 Leiden Institute for Brain and Cognition (LIBC), Leiden, The Netherlands

**Keywords:** Affect, Attention, Great ape, Touchscreen, Social cognition, Emotion perception

## Abstract

Attention may be swiftly and automatically tuned to emotional expressions in social primates, as has been demonstrated in humans, bonobos, and macaques, and with mixed evidence in chimpanzees, where rapid detection of emotional expressions is thought to aid in navigating their social environment. Compared to the other great apes, orangutans are considered semi-solitary, but still form temporary social parties in which sensitivity to others’ emotional expressions may be beneficial. The current study investigated whether implicit emotion-biased attention is also present in orangutans (*Pongo pygmaeus*). We trained six orangutans on the dot-probe paradigm: an established paradigm used in comparative studies which measures reaction time in response to a probe replacing emotional and neutral stimuli. Emotional stimuli consisted of scenes depicting conspecifics having sex, playing, grooming, yawning, or displaying aggression. These scenes were contrasted with neutral scenes showing conspecifics with a neutral face and body posture. Using Bayesian mixed modeling, we found no evidence for an overall emotion bias in this species. When looking at emotion categories separately, we also did not find substantial biases. We discuss the absence of an implicit attention bias for emotional expressions in orangutans in relation to the existing primate literature, and the methodological limitations of the task. Furthermore, we reconsider the emotional stimuli used in this study and their biological relevance.

**Supplementary Information:**

The online version contains supplementary material available at 10.1007/s42761-022-00158-x.

Salient stimuli attract attention in humans (Compton, 2003) and non-human primates (hereafter: primates) appear to share this tendency. Such attention biases are typically shaped by evolutionary pressures, as they are important for survival. Reported attention biases include the rapid detection not only of threatening stimuli, such as poisonous animals (Hopper et al., 2021; Masataka et al., 2018; Shibasaki & Kawai, 2009) or predators (Laméris et al., 2022), but also of emotionally valent stimuli (Van Rooijen et al., 2017). Based on evolutionary theories, the latter should especially be the case for social species, for whom the fast detection and recognition of such stimuli triggers corresponding behavioral responses which are thought to aid individuals in navigating their social environment (Van Rooijen et al., 2017; Vuilleumier, 2005). Namely, emotional expressions can inform group members about the expresser’s internal state and potential future behavior (Waller et al., 2017).

Despite that primates use a range of emotional expressions that are comparable between species, their use and function may differ (Kret et al., 2020), which is possibly driven by the socio-ecological environment of the species (Dobson, 2012). Bonobos (*Pan paniscus*), for example, prevent conflict with sexual interactions, play and grooming activities (Furuichi, 2011; Palagi & Norscia, 2013), and console individuals in distress (Clay & De Waal, 2013). In parallel, bonobos show an attention bias towards affiliative scenes, such as grooming and sexual activities (Kret et al., 2016) and play faces (Laméris et al., 2022). In contrast, rhesus macaques (*Macaca mulatta*) and long-tailed macaques (*Macaca fascicularis*) are considered despotic (Matsumura, 1999; Thierry, 1985) and show biased attention for threatening faces of conspecifics (Cassidy et al., 2021; King et al., 2012; Lacreuse et al., 2013; Parr et al., 2013). Important to note, however, is the absence of an attentional bias towards emotionally-salient cues in chimpanzees (*Pan troglodytes*; Kret et al., 2018; Wilson & Tomonaga, 2018). This may be related to methodological differences between studies, but also other factors such as influences of current affective states (Bethell et al., 2012; Cassidy et al., 2021) and life experiences (Leinwand et al., 2022; Puliafico & Kendall, 2006) that may modulate attention bias. Nevertheless, there is a large body of literature on biased attention towards emotions (Van Rooijen et al., 2017) and findings seem to suggest that general attention biases reflect the socio-biology of the species. This makes it interesting to study these biases in a range of species with different social structures, leading to evolutionary insights in emotion perception.

Orangutans (*Pongo* spp.) are phylogenetically close to humans and in the wild live in complex but loose social communities. Compared to the other great apes, orangutans do not form stable social groups (apart from mother-infant groupings) and live a semi-solitary existence (Delgado & Van Schaik, 2000; Galdikas, 1985; Mitra Setia et al., 2009; Roth et al., 2020; Singleton et al., 2009; Van Schaik, 1999). Their social structure is highly variable with close-range affiliations depending on sex, age, reproductive state, social status, and ecological determinants. They, nonetheless, form temporary social parties for mating opportunities, socializations for their infants and protection from male coercion. As such, orangutans show a range of expressions and behaviors potentially indicating a sensitivity to emotions (e.g., Davila-Ross et al., 2008; Laméris et al., 2020; Pritsch et al., 2017; van Berlo et al., 2020). Given their social organization, as compared to the other great apes, orangutans are an interesting model to investigate the evolutionary roots of emotion-biased attention.

Here, we investigate whether implicit emotion-biased attention is present in orangutans using the dot-probe paradigm, a suitable paradigm for comparative studies (Van Rooijen et al., 2017). In this task, two stimuli are simultaneously, and briefly (300 ms), presented to individuals on opposite sides of a touchscreen. Stimuli are two photographs, in which a neutral expression is paired with an emotional expression, although other pairings are possible as long as the two stimuli compete for attentional resources. After the brief presentation of the two stimuli, a probe emerges on the location of either the emotional stimulus (i.e., the congruent condition) or the neutral stimulus (i.e., the incongruent condition). As a consequence, attention will automatically be drawn to the most salient stimulus. This attention bias is resulting in faster reaction times in the congruent condition, i.e., to the probe replacing the stimulus that caught their attention, whereas slower reaction times indicate that attention was shifted from the other location (i.e., the less-salient stimulus or incongruent condition). As such, the dot-probe paradigm allows to investigate the implicit attentional processes involved in emotion perception.

Based on our current knowledge of facial and bodily expressions in orangutans, and their putative relevance within their social structure, we predict that orangutans show attention biases towards emotional scenes, although selectively. Currently, we know very little about which specific emotional categories are relevant to orangutans, but take prior work as a starting point (see Kret et al., 2016), who used categories such as grooming, sex, play and yawning. Orangutans use play faces flexibly and possibly intentionally (Waller et al., 2015); hence, we expect orangutans to show a bias for playful scenes. Previously, an attention bias towards grooming, sexual interactions, and yawning was reported in bonobos using a similar paradigm (Kret et al., 2016). However, although the socio-behavioral repertoire of orangutans is somewhat similar to that of other apes, orangutans affiliate less frequently. For example, bonobos use sex to maintain social bonds (De Waal, 1988), whereas orangutans do not. Thus, we expect that orangutans may show a bias for grooming, but not for sexual scenes. Moreover, as orangutans are known to pucker their lips to produce kiss-squeaks when agitated (Hardus et al., 2009), orangutans may show an attention bias towards displays of agitation. Lastly, we expect to find a bias for yawning scenes, in line with previous findings in humans and bonobos (Kret et al., 2016; Kret & Van Berlo, 2021). Although not necessarily an emotional expression, yawning is highly contagious, also in orangutans (van Berlo et al., 2020). It has been proposed that it may synchronize vigilance levels between individuals (Gallup & Gallup, 2007; Miller et al., 2012); thus, its rapid detection may be beneficial in threatening situations.

## Method

### Subjects and housing

Six Bornean orangutans (*Pongo pygmaeus*, four female and two males; mean age = 16.2 years; range = 6–36 years old), at Apenheul Primate Park (the Netherlands), participated in the current study (Table 1). The animals were part of a population of 9 orangutans housed in a building consisting of four indoor enclosures that were each connected to outdoor islands. The orangutans were typically housed in 3–4 subgroups, and group composition was regularly changed with the aim to mimic the natural social structure of orangutans in which they form temporary parties but no stable social groups. Some individuals never shared enclosures to avoid conflict (e.g., the two adult males).

**Table 1:** Subject information

Name	Sex	Birth year (age at the start of the study)
Baju^a^	Male	2015 (6)
Binti	Female	2000 (18)
Dayang	Female	2005 (13)
Kawan^a^	Male	2010 (11)
Samboja	Female	2005 (13)
Sandy	Female	1982 (36)

^a^These individuals were later added to the sample

All orangutans were naïve to touchscreen training at the start of this study. Between February 2017 and June 2017, and between October 2017 and February 2018, we initially trained four individuals successfully on the dot-probe paradigm. We had the opportunity to train an additional individual (Kawan) between June 2019 and February 2020. Another individual (Baju) sporadically joined training sessions during this period and showed immediate high accuracy scores. Although this individual did not go through different training stages, he was included in this study as he reached the inclusion criteria (as described below). Touchscreen sessions were conducted between February and April in 2018 for the first four individuals and in February 2021 for the remaining two individuals in an off-exhibit enclosure, and participation was completely voluntarily. During training and testing, orangutans had the opportunity to be surrounded by conspecifics and were thus not separated from other individuals. Nevertheless, orangutans were trained to complete the touchscreen task alone. Sessions were paused whenever another orangutan interrupted. Sessions were furthermore conducted using positive reinforcement training, using quarter pieces of hazelnuts for the initial four subjects and sunflower seeds for the two individuals that were later included, and conformed to the guidelines of the Ex-situ Program (EEP), formulated by the European Association of Zoos and Aquaria (EAZA) as well as to the guidelines formulated by Apenheul Primate Park. The test sessions of the two additional subjects were conducted following a strict COVID-protocol.

### Apparatus

All touchscreen sessions were conducted using E-Prime on a TFT-19-OF1 Infrared touchscreen (19″, 1280 × 1024 pixels). The touchscreen setup was encased in a custom-made setup which was incorporated in the orangutans’ enclosure. The researchers controlled the sessions on a laptop connected to the touchscreen setup and could monitor the orangutans’ responses on the touchscreen through a livestream with a camera that was built in the enclosure behind the orangutan. This footage was stored and later used to code the test sessions for outliers and the orangutans’ behavior. Correct responses were rewarded with small food items which were manually delivered through a PVC chute on a 100% fixed reinforcement ratio. The researcher was positioned behind the setup which prevented visual contact between the orangutans and researchers.

### Stimuli

Socio-emotional stimuli used during this study were sourced from the Internet or from personal photo libraries. Images were full-color, resized to 330 × 400 pixels and depicted unfamiliar orangutans in either neutral or emotional scenes. Neutral scenes included individuals that were resting, locomoting and showing a neutral expression. Based on previous work (Kret et al., 2016), we defined five emotional categories: *Display*, *Grooming*, *Play*, *Sex*, and *Yawn* (Table 2; Table ESM1). We reasoned to use scenes as emotional expressions consist of a combination of facial and bodily cues which together convey information about an individuals’ state and intentions (De Gelder et al., 2010). Using such stimuli may therefore be biologically more relevant than isolated cues (Kano & Tomonaga, 2010). To avoid the potential effect of predicted confounding factors, we then matched neutral and emotional stimuli according to the number of individuals present, the presence of juveniles or flanged males, and other low-level features such as luminance and contrast levels (Kano et al., 2012). Ideally, we would have included more categories such as individuals in distress or involved in agonistic interactions, but were unable to source enough stimuli.

**Table 2:** Stimulus categories used in this study, together with the number of images per category and mean valence and intensity scores

Category	Description	*n*	Mean valence (*SD*)	Mean intensity (*SD*)
Display	Kiss-squeak expression	31	2.90 (0.45)	3.58 (0.46)
Grooming	Two or more individuals engaging in grooming activities	35	5.50 (0.29)	3.30 (0.35)
Play	Solitary or social play, with relaxed open mouth	48	5.77 (0.37)	3.97 (0.36)
Sex	Mating, sexual inspection	28	5.52 (0.42)	3.83 (0.40)
Yawn	Wide open mouth, with or without canine visible	48	3.79 (0.30)	3.24 (0.41)
Neutral	Resting, locomotion, without apparent facial expressions	190	4.12 (0.32)	1.81 (0.37)

Seven people (two caretakers and five primatologists [including three authors]) rated these images on a 7-point Likert scale in terms of their valence (ranging from 1 = very negative to 7 = very positive, with 4 = neutral) and intensity (ranging from 1 = not intense at all to 7 = very intense). We calculated intraclass correlations for valence and intensity ratings using a two-way mixed model and a consistency definition. The raters showed a good intraclass correlation, ICC(3,k)_valence_ = .89; ICC(3,k)_intensity_ = .87; Table 2. The *Display* and *Yawn* category were rated as relatively negative, compared to the *Neutral* category, whereas the *Grooming*, *Play*, and *Sex* category were rated as more positive. Furthermore, the emotional stimuli were all rated as more intense than the neutral stimuli (Table ESM2–3).

### Procedure

**Figure 1:**
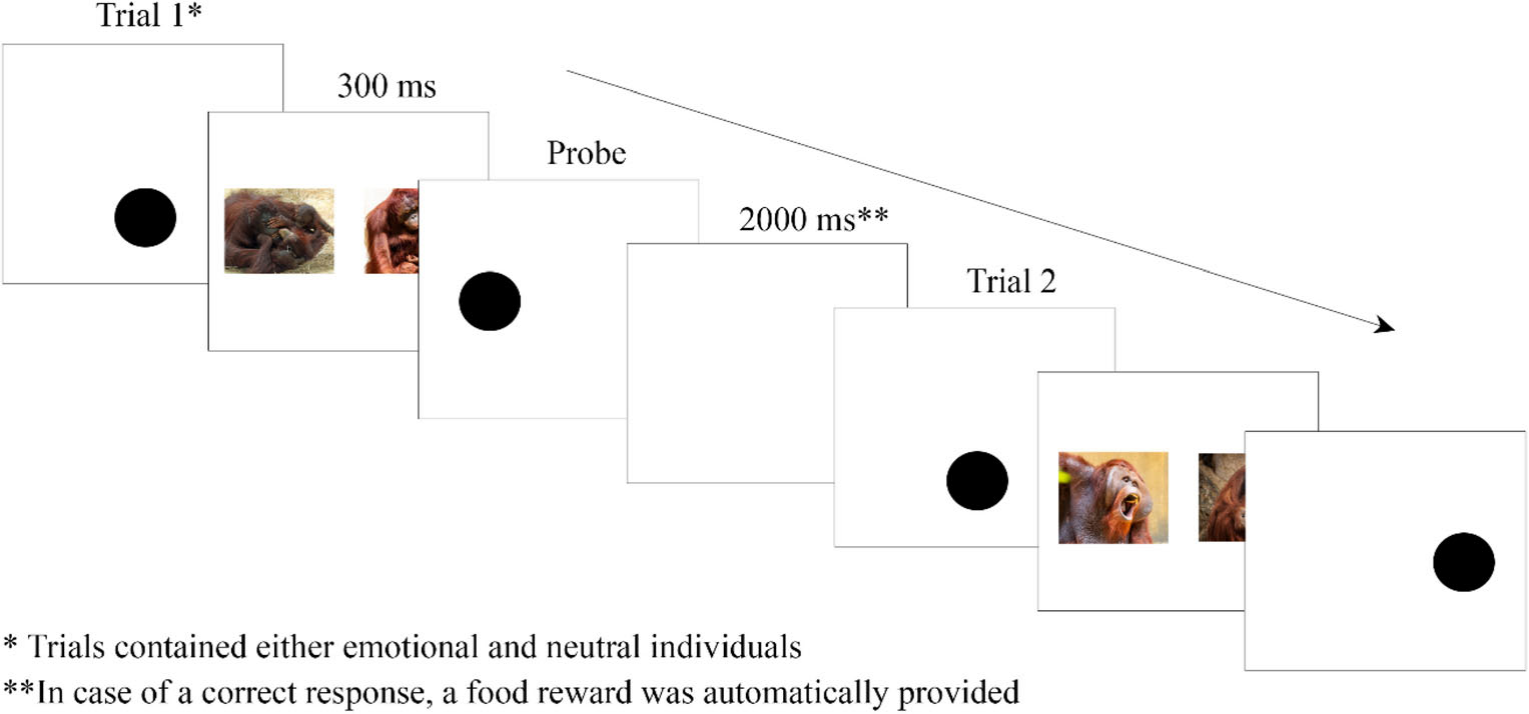
Trial outline of the dot-probe task

Because the orangutans never worked on touchscreens before, we followed step 1–6 from the training protocol described in the supplements of Kret et al. (2016) for the dot-probe paradigm. In summary, we first habituated the orangutans to the presence of the touchscreen by rewarding them when they approached the screen and used vocal appraisal. We then presented a large black dot on the screen and rewarded the individuals if they touched the screen at any location. Once the orangutans were sufficiently conditioned on the association between touching the screen and receiving a small food reward, we gradually reduced the size of the dot until reaching the final size which was used during the study (200 × 200 pixels). After the orangutans reliably touched the dot, it was followed by a similar dot, on either the left or the right side of the screen. Once this step was established, we proceeded training the orangutans on the trial outline of the dot-probe task (Figure 1): The orangutans started a trial by touching a centrally presented dot (a black circle), followed by the presentation of two pictures; after 300 ms, the pictures automatically disappeared and were followed by a single probe (a black circle) replacing one of the pictures. Pictures during the training phase consisted of colored images of various animals (rabbits, sheep) or flowers. Trials were considered correct and rewarded when the orangutans correctly touched the dot and the subsequent probe and when they were attending the task for the entire trial. Initially, four orangutans reached a 80% accuracy inclusion criterium in the beginning of 2018. One additional orangutan was later trained on the dot-probe paradigm for another study in 2021 (Roth et al., in prep), and another orangutan spontaneously participated. During the dot-probe paradigm, the animals were presented with a black dot in the lower, middle part of the screen. Touching this dot initiated the trial after which two images were immediately presented side-by-side and centered on the *y*-axis on the screen for 300 ms. One of the images was a neutral stimulus and the other consisted of an emotional stimulus. After the stimuli were presented for 300 ms, they disappeared and the probe (a similar dot) appeared on either the left or right side, replacing one of the two stimuli and remained on the screen until the animal touched the probe. After an inter-trial interval of 2,000 ms, the start dot was presented again and the orangutan could initiate the next trial. The location of the stimuli on the screen and the location of the probe were counterbalanced, and the order of presentation of the emotional categories was randomized.

The orangutans were presented with 190 unique trials, and 10 repetitions to create 8 sessions of 25 trials each. Unsuccessful trials (defined in the next section) were repeated at the end of all sessions. Ultimately, each orangutan completed between 7 and 12 sessions with an average of 248 (*SD* = 46.46) trials (range = 175–300).

### Data filtering

One researcher coded all test sessions for unsuccessful trials. A second researcher coded 25% of the trials and showed a high agreement, ICC(3,k) = .94, *p* < 0.001. Unsuccessful trials were defined as trials where orangutans were not properly sitting in front of the screen, not paying attention to the screen during stimulus presentation, not pressing the probe right after onset, switched hands when pressing the probe, where other orangutans interfered with the task, or where the screen did not immediately register a touching the probe despite a touch being visible on the camera recording. We first filtered out erroneous trials and based on these criteria, 556 out of 1,488 trials (37.4%) were removed. Next, we filtered out extremely fast or slow responses. The lower exclusion criterion was RT < 200 ms; the upper criterion was determined by calculating the median absolute deviation (MAD) per subject (i.e., RT = median + 2.5 × MAD; Leys et al., 2013). This resulted in the removal of an additional 135 trials (9.1%; Table ESM2).

### Statistical analysis

All analyses were done in RStudio (version 1.4.1106; R Core Team, 2020). Using the package “brms” (Bürkner, 2017, 2018), we fitted Bayesian mixed models to assess whether orangutans show an attention bias for emotionally laden stimuli over neutral stimuli and whether this bias is driven by pre-defined emotional categories. We chose for a Bayesian rather than frequentist approach, as it is particularly useful for small sample studies such as ours (see, e.g., Wagenmakers et al., 2008) Moreover, Bayesian analyses result in directly interpretable results. For instance, they include the 89% credible interval, which indicates the 89% probability that our effect of interest falls within the reported range (McElreath, 2018). This contrasts with the confidence interval interpretation, which only allows for making indirect inferences about the true estimate falling within a specific range (Hespanhol et al., 2019). The prime number 89 is different from the conventional 95% confidence interval in a frequentist approach in order to avoid unconscious hypothesis testing (McElreath, 2018). In addition, the analysis relies on the inclusion of prior knowledge or expectations, is therefore less sensitive to type I errors, and provides more robust results in small samples (Makowski et al., 2019).

To investigate a general bias for emotional stimuli, we fitted a Bayesian mixed model using a Student-*t* distribution, with a continuous dependent variable, reaction time (ms), and *Congruence* as an independent, categorical variable (with *congruent* trials having a probe appear behind an emotional stimulus and *incongruent* trials having a probe appear behind a neutral stimulus). *Congruence* was sum-coded. Moreover, we included nested random intercepts, namely sessions (minimum of 7 and maximum of 12 per subject) nested within subjects (6). We used a weakly informative Gaussian prior for the intercept (*M* = 500, *SD* = 100) and a more conservative Gaussian prior the fixed effect (*M* = 0, *SD* = 10). Furthermore, we used the default half Student-*t* priors with 3 degrees of freedom for the random effects and residual standard deviation.

In the second model where we zoomed in on emotion categories, we fitted a Bayesian mixed (Student-*t*) model with reaction time as dependent variable and an interaction between *Congruence* and *Emotion Category* (with the categories *Sex, Play, Grooming, Yawning, Display*). *Congruence* and *Emotion Category* were sum-coded (also known as effect coding), and we included a nested random intercept (session within subject). We used the same prior settings as in the previous model (Gaussian priors for the intercept and independent variables, default half Student-*t* priors for the random effects and residual standard deviation).

To further substantiate our findings, we calculated a Bayes factor (BF) for both of our models by comparing them to an intercept-only (null) model. The BF can quantify the amount of evidence for or against a hypothesis (see, e.g., Lee & Wagenmakers, 2013). We also conducted post hoc analyses to assess the influence of various potential confounds (e.g., stimulus intensity, presence or absence of infants and flanged males); as we did not find evidence for an effect for any of these, a description of these analyses and their results can be found in the provided Supplementary Material.

To summarize the results, we report (i) the median difference between conditions; (ii) the 89% credible interval (CI); (iii) the probability of direction (pd), reflecting the certainty with which an effect goes in a specific direction (here: a faster reaction time to probes replacing emotional stimuli) and ranging between 50 and 100% (Makowski et al., 2019); and (iv) the Bayes factor.

We check the validity of our models using the WAMBS checklist (Depaoli & van de Schoot, 2017). For every model, we ran 4 chains and 40,000 iterations (including 2,000 warm-up iterations). Model convergence was checked by inspecting trace plots, histograms of the posteriors, Gelman-Rubin diagnostics, and autocorrelation between iterations (Depaoli & van de Schoot, 2017). No divergences or excessive autocorrelations were found.

## Results

**Figure 2:**
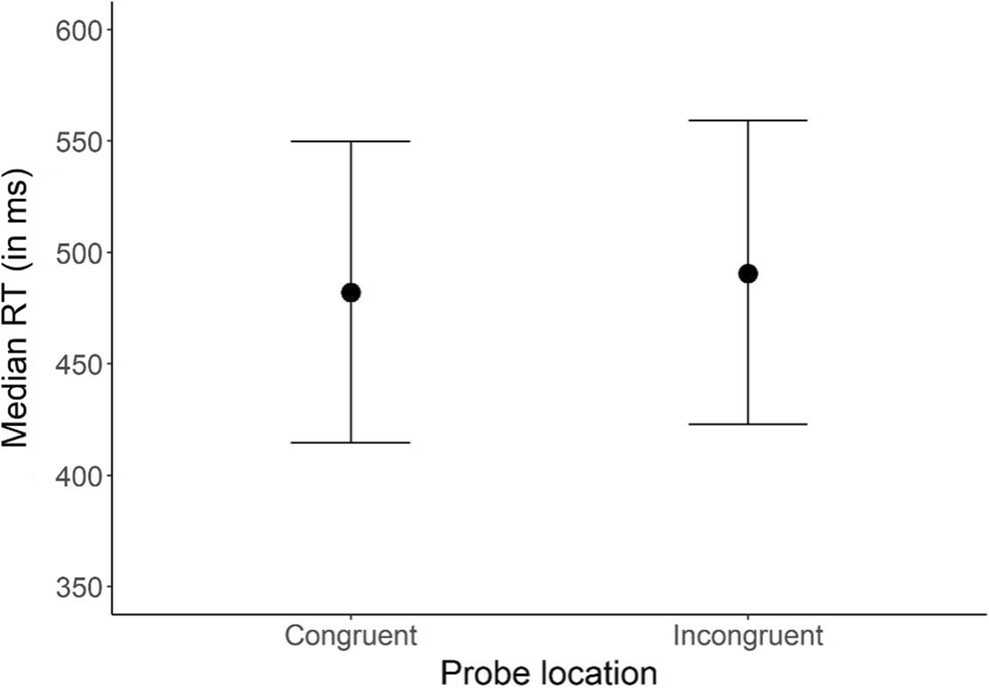
Median reaction time (in milliseconds) per probe location. Congruent trials represent trials in which the probe appeared behind an emotional stimulus, whereas in incongruent trials, the probe appeared behind a neutral stimulus. Error bars represent the 89% credible interval

**Figure 3:**
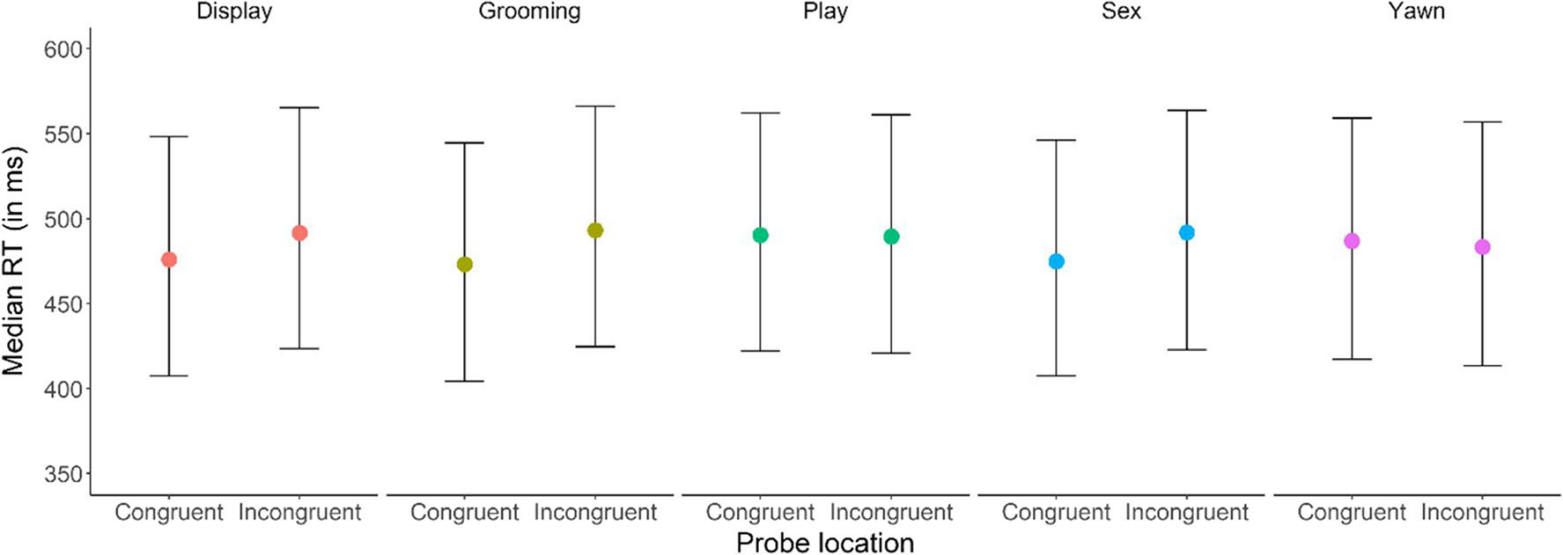
Median reaction time (in milliseconds) per emotion category and probe location. Congruent trials represent trials in which the probe appeared behind an emotional stimulus, whereas in incongruent trials, the probe appeared behind a neutral stimulus. Error bars represent the 89% credible interval

For our first model, in which we investigated a general bias for emotional stimuli over neutral stimuli, we did not find a robust effect for *Congruence* on reaction time (median difference_neutral-emotional_ = 7.70 ms, 89% CI [−10.94 to 26.30], pd = 0.75; see Table 3 and Figure 2). This conclusion can be drawn based on the 89% credible interval (CI), which contains values indicating a positive as well as negative difference between reaction times on probes appearing behind emotional and neutral stimuli. The pd indicates a 75% certainty that the effect is in the direction that we expect (i.e., orangutans have a bias for emotional stimuli), but it does not inform us about how plausible the null-hypothesis (i.e., no difference between emotional and neutral stimuli) is. To find the strength of evidence for our null finding, we computed the Bayes factor in favor of the null-hypothesis over the alternative hypothesis (BF_01_) and found BF_01_ = 1.35, indicating anecdotal evidence for the null hypothesis (Lee & Wagenmakers, 2013). As such, orangutans did not show a bias for emotional over neutral stimuli in our study, but more data are needed to draw definitive conclusions. In the second model, where we looked at specific emotion categories, we again found no robust evidence for an attention bias for specific emotions (Yawn: median difference_neutral-emotional_ = −2.95 ms, 89% CI [−41.81 to 36.17], pd = 0.45; Display: median difference_neutral-emotional_ = 15.67 ms, 89% CI [−16.57 to 48.03], pd = 0.78; Grooming: median difference_neutral-emotional_ = −2.02 ms, 89% CI [−32.32 to 27.89], pd = 0.46; Play: median difference_neutral-emotional_ = 20.58 ms, 89% CI [−11.82 to 52.62], pd = 0.84; Sex: median difference_neutral-emotional_ = 12.57, 89% CI [−20.37 to 45.36], pd = 0.73; see Table 4 and Figure 3; also see Table ESM3 and Figure ESM3 for individual results). Calculation of the subsequent Bayes factor indicated moderate evidence for the null-hypothesis (BF_01_ = 4.79; Lee & Wagenmakers, 2013).

**Table 3:** Model output for model 1 (general emotion bias)

Parameter	Median estimate	*SD*	89% CI (lower)	89% CI (upper)
Intercept	489.65	42.14	423.88	556.28
Congruence (probe behind emotion)	−3.86	5.82	−13.15	5.4
Random effects				
SD (subject)	109.26	44.40	59.30	188.17
SD (subject:session)	59.23	11.62	41.23	78.22
*N*_obs_ = 798				
*N*_subj_ = 6				

*Congruence* is sum-coded

**Table 4:** Model output for model 2 (emotion category bias)

Parameter	Median estimate	*SD*	89% CI (lower)	89% CI (upper)
Intercept	489.40	42.18	420.02	556.42
Congruence (probe behind emotion)	−4.37	5.85	−13.71	4.99
Display	0.22	7.98	−12.54	12.95
Grooming	−1.33	7.97	−14.08	11.38
Play	3.91	7.60	−8.23	16.06
Sex	−3.48	8.06	−16.35	9.39
Congruence (emotion):Display	−3.47	7.99	−16.30	9.29
Congruence (emotion):Grooming	−5.88	7.99	−18.61	6.87
Congruence (emotion):Play	5.43	7.61	−6.75	17.61
Congruence (emotion):Sex	−1.89	8.10	−14.78	11.08
Random effects				
SD (subject)	109.26	43.18	59.52	187.64
SD (subject:session)	59.01	11.65	41.05	78.03
*N*_obs_ = 798				
*N*_subj_ = 6				

*Congruence* and *Emotion Category* are sum-coded

## Discussion

The current study investigated whether orangutans show an attention bias towards emotional stimuli. Contrary to our predictions, the orangutans in our sample did not show an attentional bias to emotions, nor towards specific emotional categories. However, more data are needed to make a decisive conclusion whether these effects are truly absent in orangutans. Below, we discuss several reasons for our findings.

We applied the dot-probe paradigm, a well-validated paradigm in humans (but see Puls & Rothermund, 2018), and a promising tool for comparative studies (Van Rooijen et al., 2017). Several studies have successfully used the paradigm with primates (Cassidy et al., 2021; King et al., 2012; Kret et al., 2016; Lacreuse et al., 2013; Leinwand et al., 2022; Parr et al., 2013), although, like our current study, not all report significant results (see e.g., Kret et al., 2018; Wilson & Tomonaga, 2018 for null-findings in chimpanzees). However, several methodological parameters may explain these inconsistencies.

Stimulus presentation duration can determine what attentional process is measured and therefore affects study outcome. Long stimulus exposure may result in the involvement of the prefrontal cortex and attentional control (Cisler & Koster, 2010; Weierich et al., 2008), thus not measuring implicit attention bias (Cassidy et al., 2021). To measure implicit attention to specific stimuli, presentation times have to be short enough to prevent saccades, which occur around the 250-ms mark in humans as well as other primates, including great apes (Fuchs, 1967; Kano & Tomonaga, 2011). As such, a stimulus presentation duration of around 250–300 ms is an appropriate threshold for stimuli being clearly (supraliminally) visible (Ben-Haim et al., 2021). To be in line with previous studies (Kret et al., 2016, 2018) and recommendations stemming from the human literature (Petrova et al., 2013), we used a stimulus presentation duration of 300 ms, which is most likely to target implicit stages of attention. Given the existing evidence, we have no reason to believe that our presentation time was somehow insufficient to measure an attentional bias for emotional expressions in orangutans. Nevertheless, most studies on the visual system of primates have been conducted in monkeys, and only very few studies have thus far compared gaze patterns in different great ape species (see e.g., Kano et al., 2012; Kano & Tomonaga, 2011). As such, more work is needed to pinpoint potential species-specific characteristics in visual processing.

Moreover, stimulus pairing may influence test outcomes (Van Rooijen et al., 2017). Emotional stimuli can be paired with scrambled stimuli (Parr et al., 2013), neutral images without conspecifics (Kret et al., 2016), neutral stimuli (King et al., 2012; Kret et al., 2018; Lacreuse et al., 2013; Leinwand et al., 2022; Wilson & Tomonaga, 2018), or other emotional stimuli. Differences in saliency or low-level features between the emotional and paired stimulus potentially influence detectability of biases towards the stimuli of interest. For instance, Wilson and Tomonaga (2018) tested chimpanzees and paired threatening facial expressions with scrambled images, for which they found an attention bias, and additionally paired threatening stimuli with neutral stimuli, for which no evidence of a bias was found. As we only included emotion-neutral pairings in our study, future work investigating emotion-biased attention in orangutans could include different types of pairings to disentangle effects of, e.g., seeing (familiar or unfamiliar) conspecifics, scrambled images, or neutral images without conspecifics to rule out potential effects of low-level features (Tomonaga & Imura, 2015).

Possibly, the used stimuli were not biologically relevant enough for the orangutans, or still-images do not adequately represent the saliency of the actual expressions. Considering that we selected our emotional categories based on previous findings (King et al., 2012; Kret et al., 2016; Parr et al., 2013) and on work indicating orangutans have a sensitivity to emotional expressions of conspecifics (Davila-Ross et al., 2008; Pritsch et al., 2017), we deem this unlikely. Simultaneously, we presented a limited number of categories and other emotional expressions might induce attention biases. For instance, an eye-tracking study with Sumatran orangutans has shown that they looked longer at emotional stimuli compared to neutral ones, specifically looking longer at the silent bared-teeth face, but not at the bulging lip face (Pritsch et al., 2017). Moreover, following Kret et al. (2016), multiple experts classified the stimuli in terms of their emotional valence and intensity and showed high inter-rater reliability, suggesting that the ratings of the used categories are trustworthy. We encourage future studies to include more emotional categories, including, for example, silent bared-teeth face or bulging lip face.

Alternatively, it is possible that orangutans simply do not attend to emotional stimuli automatically. Implicit attention biases are theoretically expected to be the strongest in highly social species where the rapid detection and recognition of another’s emotional expression is needed for appropriate responses (Spoor & Kelly, 2004). Orangutans lead semi-solitary lives, and hence, it might not be important for them to be implicitly sensitive to other’s emotions, while this is arguably beneficial for obligate group-living species (see e.g., findings by Lewis et al., 2021). In contrast, emotional expressions of unknown individuals might be more relevant as such individuals pose a potential higher likelihood of threat or unpredictability (Campbell & De Waal, 2011). Our results do not give clear evidence to either confirm or reject this hypothesis, although it seems unlikely that orangutans do not implicitly attend to emotional stimuli. For example, the flexible production of play faces (Waller et al., 2015) and rapid mimicry (Davila-Ross et al., 2008) suggest that orangutans are able to quickly recognize and respond to such facial expressions. Play facial expressions are, however, arguably more relevant for juveniles, potentially explaining why we did not find a bias for such stimuli in our sample as the majority were adults (5 out of 6 individuals). Equally for other emotional categories, it is possible that individual characteristics of the subjects, such as sex (Howarth et al., 2021), age range, temperamental predispositions, current affective states (Bethell et al., 2012; Cassidy et al., 2021), and life experiences (Leinwand et al., 2022; Puliafico & Kendall, 2006), influenced the relevance of emotional scenes, therefore limiting the interpretability of our results. For instance, orangutan aggregation patterns are highly variable, and sex-specific patterns for example may differ between sites (Galdikas, 1985; Roth et al., 2020), which could influence sex-specific effects on attention. Testing for such individual differences is beyond the scope of the current sample size, but visual inspection of the results per individual showed that the absence of evidence for attentional biases was consistent across our sampled individuals.

Moreover, characteristics about the individuals depicted on the stimuli, such as facial characteristics, may have obfuscated the effect of emotion on attention. It has previously been reported that orangutans look more at the eyes of juveniles than to adult eyes (Kano et al., 2012). The lighter coloring around the eyes of juvenile orangutans and flanged cheeks of males may present conspicuous facial features that are attractive. To control for these characteristics, we carefully paired emotional and neutral stimuli and took into account if the expresser was a juvenile or flanged male. We tested if the presence of juveniles or flanged males on either the probe, non-probe or both influenced the reaction times, but found no such effect. Hence, we can only conclude that the absence of a bias for emotional stimuli was not modulated by these facial features.

Interestingly, we did not find a bias for yawning scenes. Bonobos previously showed a strong attention bias for yawning (while controlling for canine visibility; Kret et al., 2016). Indeed, yawns are contagious between bonobos and contagion is stronger between kin and friends or when expressed by a high ranking group member (Demuru & Palagi, 2012; Massen et al., 2012; Palagi et al., 2014). In our earlier work, we provided experimental evidence for yawn contagion in orangutans (van Berlo et al., 2020), although this effect was independent of the familiarity of the stimulus individual. Yawning potentially facilitates thermoregulation of the brain (Massen et al., 2021), where cooling may promote vigilance. The contagiousness of yawning within a group may consequently synchronize vigilance (Bower et al., 2012; Gallup & Gallup, 2007) and thus may be beneficial to quickly attend to. Given that yawning is a dynamic facial expression, the still images of yawns in our study may lack crucial information for orangutans to elicit an attention bias.

The absence of a bias for other emotional stimuli, such as the display (i.e., kiss-squeak), may be explained simply that the associated facial expression is more like a by-product, rather than a signal in and of itself. Kiss-squeaks are mostly produced in response to predators or other orangutans (Hardus et al., 2009) and predominantly function as an auditory signal (Lameira et al., 2013), hence explaining why an implicit attention bias is absent. A recent study showed that a visual attention bias can be strengthened by including congruent auditory signals (e.g., hearing an alarm call when viewing a predator; Sato et al., 2021). This method provides an interesting way to complement emotion-biased attention in the future.

In conclusion, we found no convincing evidence for implicit attention biases for scenes depicting display, grooming, sex, play, or yawning and addressed a number of methodological parameters that may explain these findings. Future studies could focus on exploring attention to a wider range of social and emotional scenes further, for instance, by including auditory signals. Individual factors might have influenced our results, and we recommend future studies to take this into account when possible. Orangutans remain interesting study subjects for investigating emotion-biased attention, given their unique social structure. We therefore encourage future studies to investigate both implicit and explicit attention processing for emotional stimuli.

## Supplementary Information

ESM 1 (DOCX 391 kb)

## Additional Information

### Availability of Data and Material

All data that are associated with this paper and used to conduct the analyses are accessible on the archiving platform Dataverse NL through the last author’s institute, Leiden University: 10.34894/LG6M34. For copyright reasons, all stimuli are available upon request via Dataverse NL.

### Code Availability

All software and code adjustments that are associated with this paper are openly accessible on the archiving platform Dataverse NL through the last author’s institute, Leiden University: 10.34894/LG6M34.

## References

[CR1] Ben-Haim MS, Monte OD, Fagan NA, Dunham Y, Hassin RR, Chang SWC, Santos LR (2021). Disentangling perceptual awareness from nonconscious processing in rhesus monkeys (Macaca mulatta). Proceedings of the National Academy of Sciences of the United States of America.

[CR2] Bethell EJ, Holmes A, MacLarnon A, Semple S (2012). Evidence that emotion mediates social attention in Rhesus Macaques. PLoS ONE.

[CR3] Bower S, Suomi SJ, Paukner A (2012). Evidence for kinship information contained in the rhesus macaque (*Macaca mulatta*) face. Journal of Comparative Psychology.

[CR4] Bürkner PC (2017). brms: an R package for Bayesian multilevel models using Stan. Journal of Statistical Software.

[CR5] Bürkner PC (2018). Advanced Bayesian multilevel modeling with the R package brms. R Journal.

[CR6] Campbell MW, De Waal FBM (2011). Ingroup-outgroup bias in contagious yawning by chimpanzees supports link to empathy. PLoS ONE.

[CR7] Cassidy, L. C., Bethell, E. J., Brockhausen, R. R., Boretius, S., Treue, S., & Pfefferle, D. (2021). The dot-probe attention bias task as a method to assess psychological well-being after anesthesia: a study with adult female long-tailed macaques (Macaca fascicularis). *European Surgical Research*. 10.1159/000521440 10.1159/000521440 PMC9909723 34915502

[CR8] Cisler JM, Koster EHW (2010). Mechanisms of attentional biases towards threat in anxiety disorders: an integrative review. Clinical Psychology Review.

[CR9] Clay Z, De Waal FBM (2013). Bonobos respond to distress in others: consolation across the age spectrum. PLoS ONE.

[CR10] Compton RJ (2003). The interface between emotion and attention: a review of evidence from psychology and neuroscience. Behavioral and Cognitive Neuroscience Reviews.

[CR11] Davila-Ross, M., Menzler, S., & Zimmermann, E. (2008). Rapid facial mimicry in orangutan play. *Biology Letters, 4*(1), 27–30. 10.1098/rsbl.2007.0535 PMC2412946 18077238

[CR12] De Gelder B, Van den Stock J, Meeren HKM, Sinke CBA, Kret ME, Tamietto M (2010). Standing up for the body. Recent progress in uncovering the networks involved in the perception of bodies and bodily expressions. Neuroscience and Biobehavioral Reviews.

[CR13] De Waal FBM (1988). The communicative repertoire of captive bonobos (*Pan paniscus*), compared to that of chimpanzees. Behaviour.

[CR14] Delgado RA, Van Schaik CP (2000). The behavioral ecology and conservation of the orangutan (*Pongo pygmaeus*): a tale of two islands. Evolutionary Anthropology.

[CR15] Demuru E, Palagi E (2012). In bonobos yawn contagion is higher among kin and friends. PLoS ONE.

[CR16] Depaoli S, van de Schoot R (2017). Improving transparency and replication in Bayesian statistics: the WAMBS-Checklist. Psychological Methods.

[CR17] Dobson SD (2012). Coevolution of facial expression and social tolerance in macaques. American Journal of Primatology.

[CR18] Fuchs AF (1967). Saccadic and smooth pursuit eye movements in the monkey. The Journal of Physiology.

[CR19] Furuichi T (2011). Female contributions to the peaceful nature of bonobo society. Evolutionary Anthropology.

[CR20] Galdikas BMF (1985). Orangutan sociality at Tanjung Puting. American Journal of Primatology.

[CR21] Gallup, A. C., & Gallup, G. G. (2007). Yawning as a brain cooling mechanism: nasal breathing and forehead cooling diminish the incidence of contagious yawning. *Evolutionary Psychology, 5*(1). 10.1177/147470490700500109

[CR22] Hardus ME, Lameira AR, Van Schaik CP, Wich SA (2009). Tool use in wild orang-utans modifies sound production: a functionally deceptive innovation?. Proceedings of the Royal Society B: Biological Sciences.

[CR23] Hespanhol L, Vallio CS, Costa LM, Saragiotto BT (2019). Understanding and interpreting confidence and credible intervals around effect estimates. Brazilian Journal of Physical Therapy.

[CR24] Hopper LM, Allritz M, Egelkamp CL, Huskisson SM, Jacobson SL, Leinwand JG, Ross SR (2021). A comparative perspective on three primate species’ responses to a pictorial emotional stroop task. Animals.

[CR25] Howarth ERI, Kemp C, Thatcher HR, Szott ID, Farningham D, Witham CL, Holmes A, Semple S, Bethell EJ (2021). Developing and validating attention bias tools for assessing trait and state affect in animals: a worked example with *Macaca mulatta*. Applied Animal Behaviour Science.

[CR26] Kano F, Call J, Tomonaga M (2012). Face and eye scanning in Gorillas (*Gorilla gorilla*), orangutans (*Pongo abelii*), and humans (*Homo sapiens*): unique eye-viewing patterns in humans among hominids. Journal of Comparative Psychology.

[CR27] Kano F, Tomonaga M (2010). Attention to emotional scenes including whole-body expressions in chimpanzees (*Pan troglodytes*). Journal of Comparative Psychology.

[CR28] Kano F, Tomonaga M (2011). Species difference in the timing of gaze movement between chimpanzees and humans. Animal Cognition.

[CR29] King HM, Kurdziel LB, Meyer JS, Lacreuse A (2012). Effects of testosterone on attention and memory for emotional stimuli in male rhesus monkeys. Psychoneuroendocrinology.

[CR30] Kret ME, Jaasma L, Bionda T, Wijnen JG (2016). Bonobos (*Pan paniscus*) show an attentional bias toward conspecifics’ emotions. Proceedings of the National Academy of Sciences.

[CR31] Kret ME, Muramatsu A, Matsuzawa T (2018). Supplemental material for emotion processing across and within species: a comparison between humans (*Homo sapiens*) and chimpanzees (*Pan troglodytes*). Journal of Comparative Psychology.

[CR32] Kret ME, Prochazkova E, Sterck EHMM, Clay Z, Kret ME (2020). Emotional expressions in human and non-human great apes. Neuroscience and Biobehavioral Reviews.

[CR33] Kret ME, Van Berlo E (2021). Attentional bias in humans toward human and bonobo expressions of emotion. Evolutionary Psychology.

[CR34] Lacreuse A, Schatz K, Strazzullo S, King HM, Ready R (2013). Attentional biases and memory for emotional stimuli in men and male rhesus monkeys. Animal Cognition.

[CR35] Lameira, A. R., Hardus, M. E., Nouwen, K. J. J. M., Topelberg, E., Delgado, R. A., Spruijt, B. M., Sterck, E. H. M., Knott, C. D., & Wich, S. A. (2013). Population-specific use of the same tool-assisted alarm call between two wild orangutan populations (*Pongo pygmaeus wurmbii*) indicates functional arbitrariness. *PLoS ONE, 8*(7). 10.1371/journal.pone.0069749 10.1371/journal.pone.0069749 PMC3702587 23861981

[CR36] Laméris DW, Van Berlo E, Sterck EHM, Bionda T, Kret ME (2020). Low relationship quality predicts scratch contagion during tense situations in orangutans (*Pongo pygmaeus*). American Journal of Primatology.

[CR37] Laméris DW, Verspeek J, Eens M, Stevens JMG (2022). Social and nonsocial stimuli alter the performance of bonobos during a pictorial emotional Stroop task. American Journal of Primatology.

[CR38] Lee MD, Wagenmakers E-J (2013). Bayesian cognitive modeling. Bayesian Cognitive Modeling: a Practical Course.

[CR39] Leinwand JG, Fidino M, Ross SR, Hopper LM (2022). Familiarity mediates apes’ attentional biases toward human faces. Proceedings of the Royal Society B: Biological Sciences.

[CR40] Lewis LS, Kano F, Stevens JMG, DuBois JG, Call J, Krupenye C (2021). Bonobos and chimpanzees preferentially attend to familiar members of the dominant sex. Animal Behaviour.

[CR41] Leys C, Ley C, Klein O, Bernard P, Licata L (2013). Detecting outliers: do not use standard deviation around the mean, use absolute deviation around the median. Journal of Experimental Social Psychology.

[CR42] Makowski D, Ben-Shachar MS, Chen SHA, Lüdecke D (2019). Indices of effect existence and significance in the Bayesian framework. Frontiers in Psychology.

[CR43] Masataka N, Koda H, Atsumi T, Satoh M, Lipp OV (2018). Preferential attentional engagement drives attentional bias to snakes in Japanese macaques (*Macaca fuscata*) and humans (*Homo sapiens*). Scientific Reports.

[CR44] Massen JJM, Hartlieb M, Martin JS, Leitgeb EB, Hockl J, Kocourek M, Olkowicz S, Zhang Y, Osadnik C, Verkleij JW, Bugnyar T, Němec P, Gallup AC (2021). Brain size and neuron numbers drive differences in yawn duration across mammals and birds. Communications Biology.

[CR45] Massen JJM, Vermunt DA, Sterck EHM (2012). Male yawning is more contagious than female yawning among chimpanzees (*Pan troglodytes*). PLoS ONE.

[CR46] Matsumura S (1999). The evolution of “egalitarian” and “despotic” social systems among macaques. Primates.

[CR47] McElreath R (2018). Statistical rethinking: a bayesian course with examples in R and stan. Statistical Rethinking: A Bayesian Course with Examples in R and Stan.

[CR48] Miller ML, Gallup AC, Vogel AR, Clark AB (2012). Auditory disturbances promote temporal clustering of yawning and stretching in small groups of budgerigars (Melopsittacus undulatus). Journal of Comparative Psychology.

[CR49] Mitra Setia, T., Delgado, R. A., Utami Atmoko, S. S., Singleton, I., & van Schaik, C. P. (2009). Social organization and male-female relationships. In S. A. Wich, S. S. Utami Atmoko, T. Mitra Setia, & C. P. van Schaik (Eds.), *Orangutans: geographic variation in behavioral ecology and conservation* (pp. 245–254). Oxford University Press. 10.5167/uzh-31343

[CR50] Palagi E, Norscia I (2013). Bonobos protect and console friends and kin. PLoS ONE.

[CR51] Palagi E, Norscia I, Demuru E (2014). Yawn contagion in humans and bonobos: emotional affinity matters more than species. PeerJ.

[CR52] Parr LA, Modi M, Siebert E, Young LJ (2013). Intranasal oxytocin selectively attenuates rhesus monkeys’ attention to negative facial expressions. Psychoneuroendocrinology.

[CR53] Petrova K, Wentura D, Bermeitinger C (2013). What happens during the stimulus onset asynchrony in the dot-probe task? Exploring the role of eye movements in the assessment of attentional biases. PLoS ONE.

[CR54] Pritsch C, Telkemeyer S, Mühlenbeck C, Liebal K (2017). Perception of facial expressions reveals selective affect-biased attention in humans and orangutans. Scientific Reports.

[CR55] Puliafico AC, Kendall PC (2006). Threat-related attentional bias in anxious youth: a review. Clinical Child and Family Psychology Review 2006 9:3.

[CR56] Puls S, Rothermund K (2018). Attending to emotional expressions: no evidence for automatic capture in the dot-probe task. Cognition and Emotion.

[CR57] R Core Team. (2020). *R: a language and environment for statistical computing. R Foundation for Statistical Computing, Vienna, Austria*. https://www.r-project.org/

[CR58] Roth, T.S., Bionda, T.R, & Kret, M.E., (in prep). No implicit attentional bias towards or preference for male secondary sexual characteristics in Bornean orangutans (*Pongo pygmaeus*). 10.1038/s41598-024-62187-9 PMC11130206 38802458

[CR59] Roth TS, Rianti P, Fredriksson GM, Wich SA, Nowak MG (2020). Grouping behavior of Sumatran orangutans (*Pongo abelii*) and Tapanuli orangutans (*Pongo tapanuliensis*) living in forest with low fruit abundance. American Journal of Primatology.

[CR60] Sato Y, Kano F, Morimura N, Tomonaga M, Hirata S (2021). Chimpanzees (Pan troglodytes) exhibit gaze bias for snakes upon hearing alarm calls. Journal of Comparative Psychology.

[CR61] Shibasaki M, Kawai N (2009). Rapid detection of snakes by japanese monkeys (*Macaca fuscata*): an evolutionarily predisposed visual system. Journal of Comparative Psychology.

[CR62] Singleton I, Knott CD, Morrogh-Bernard HC, Wich SA, Van Schaik CP, Wich SA, Utami Atmoko SS, Mitra Setia T, van Schaik CP (2009). Ranging behavior of orangutan females and social organization. Orangutans: Geographic Variation in Behavioral Ecology and Conservation.

[CR63] Spoor JR, Kelly JR (2004). The evolutionary significance of affect in groups: communication and group bonding. Group Processes & Intergroup Relations.

[CR64] Thierry B (1985). Patterns of agonistic interactions in three species of macaque (*Macaca mulatta, M. fascicularis, M. tonkeana*). Aggressive Behavior.

[CR65] Tomonaga, M., & Imura, T. (2015). Efficient search for a face by chimpanzees (*Pan troglodytes*). *Scientific Reports, 5*, 11437. 10.1038/srep11437 10.1038/srep11437 PMC4504146 26180944

[CR66] Van Berlo E, Díaz-Loyo AP, Juárez-Mora OE, Kret ME, Massen JJMM (2020). Experimental evidence for yawn contagion in orangutans (*Pongo pygmaeus*). Scientific Reports.

[CR67] Van Rooijen R, Ploeger A, Kret ME (2017). The dot-probe task to measure emotional attention: a suitable measure in comparative studies?. Psychonomic Bulletin and Review.

[CR68] Van Schaik CP (1999). The socioecology of fission-fusion sociality in Orangutans. Primates.

[CR69] Vuilleumier P (2005). How brains beware: neural mechanisms of emotional attention. Trends in Cognitive Sciences.

[CR70] Wagenmakers, E. J., Lee, M., Lodewyckx, T., & Iverson, G. J. (2008). Bayesian versus frequentist inference. In H. Hoijtink, I. Klugkist, P. A. Goelen (Eds.), *Bayesian evaluation of informative hypotheses* (pp. 181–207). Springer.

[CR71] Waller BM, Caeiro CC, Davila-Ross M (2015). Orangutans modify facial displays depending on recipient attention. PeerJ.

[CR72] Waller BM, Whitehouse J, Micheletta J (2017). Rethinking primate facial expression: a predictive framework. Neuroscience and Biobehavioral Reviews.

[CR73] Weierich MR, Treat TA, Hollingworth A (2008). Theories and measurement of visual attentional processing in anxiety. Cognition & Emotion.

[CR74] Wilson DA, Tomonaga M (2018). Exploring attentional bias towards threatening faces in chimpanzees using the dot probe task. PLoS ONE.

