# Supplementary material for: No Evidence for Biased Attention Towards Emotional Scenes in Bornean Orangutans (Pongo pygmaeus)
Source: Affect Sci. 2022 Nov 24;3(4):772–82. doi: 10.1007/s42761-022-00158-x (PMC9743850; doi:10.1007/s42761-022-00158-x)
Supplement: Supplementary file 1 — (DOCX 391 kb) [file 42761_2022_158_MOESM1_ESM.docx]

**No evidence for biased attention towards emotional scenes in Bornean orangutans (*Pongo pygmaeus*)**

Laméris, D. W.^1,2*^, Van Berlo, E.^3,4,5*^, Roth, T. S.^3,6^ & Kret, M. E.^3,7^

1. Behavioural Ecology and Ecophysiology Group, Department of Biology, Wilrijk, University of Antwerp, Antwerp, Belgium

2. Antwerp ZOO Centre for Research & Conservation (CRC), Royal Zoological Society of Antwerp (RZSA), Antwerp, Belgium
3. Cognitive Psychology Unit, Institute of Psychology, Leiden University, Leiden, the Netherlands

4. Institute for Biodiversity and Ecosystem Dynamics, University of Amsterdam, Amsterdam, the Netherlands

5. Amsterdam Brain and Cognition, University of Amsterdam, Amsterdam, the Netherlands
6. Apenheul Primate Park, Apeldoorn, the Netherlands

7. Leiden Institute for Brain and Cognition (LIBC), Leiden, the Netherlands
* Authors equally contributed to the paper

**Corresponding author:** m.e.kret@fsw.leidenuniv.nl

**ORCID:**

Laméris, D.W. 0000-0002-3096-7920

Van Berlo, E. 0000-0002-5523-7721

Roth, T.S. 0000-0002-2985-120X

Kret, M.E. 0000-0002-3197-5084

**Keywords:** Affect, Attention, Great ape, Touchscreen, Social Cognition, Emotion Perception

**Statements and Declarations**

The authors have no relevant financial or non-financial interests to disclose.

**Supplemental Methods**

Table ESM1: Stimulus information.

| Stimulus category | Description | No. individuals (SE) | No. juveniles (SE) | No. flanged males (SE) |
| --- | --- | --- | --- | --- |
| Display | Kiss squeaking expression | 1.097 (0.054) | 0.065 (0.045) | 0.387 (0.089) |
| Grooming | Two or more individuals engaging in grooming activities | 2.029 (0.029) | 0.200 (0.069) | 0.143 (0.060) |
| Play | Solitary or social play, with relaxed open mouth | 1.813 (0.057) | 1.438 (0.098) | 0.021 (0.021) |
| Sex | Mating, sexual inspection | 2.036 (0.036) | 0.000 (0.000) | 0.357 (0.092) |
| Yawn | Wide open mouth, with or without canine visible | 1.021 (0.021) | 0.167 (0.054) | 0.333 (0.069) |
| Neutral | Resting, locomotion, without apparent facial expressions | 1.568 (0.038) | 0.400 (0.050) | 0.205 (0.029) |

Table ESM2: Stimulus valence and intensity values.

| Stimulus category | Mean valence (SD) | Mean intensity (SD) |
| --- | --- | --- |
| Display | 2.90 (0.45) | 3.58 (0.46) |
| Grooming | 5.50 (0.29) | 3.30 (0.35) |
| Play | 5.77 (0.37) | 3.97 (0.36) |
| Sex | 5.52 (0.42) | 3.83 (0.40) |
| Yawn | 3.79 (0.30) | 3.24 (0.41) |
| Neutral | 4.12 (0.32) | 1.81 (0.37) |

Table ESM3: Data filtering per subject.

| Subject | Tested trials (sessions) | No. of repetitions | Excluded trials (% of the total) |
| --- | --- | --- | --- |
| Baju | 200 (8) | 10 | 89 (45%) |
| Binti | 250 (10) | 50 | 115 (46%) |
| Dayang | 300 (12) | 100 | 166 (55%) |
| Kawan | 175 (7) | 9 | 70 (40%) |
| Samboja | 295 (12) | 70 | 139 (32%) |
| Sandy | 268 (11) | 68 | 111 (41%) |

**Supplemental Results**

Table ESM4: Results per subject.

| **Subject** | **Probe location** | **N** | **Mean RT** | ***SD*** | ***SE*** | ***95% CI**** |
| --- | --- | --- | --- | --- | --- | --- |
| Sandy  Sandy | Congruent | 81 | 648.09 | 438.20 | 48.69 | 96.89 |
|  | Incongruent | 76 | 693.83 | 456.45 | 52.36 | 104.30 |
| Samboja  Samboja | Congruent | 97 | 495.42 | 309.10 | 31.38 | 62.30 |
|  | Incongruent | 59 | 502.15 | 301.90 | 39.30 | 78.68 |
| Binti  Binti | Congruent | 75 | 487.79 | 244.40 | 28.22 | 56.23 |
|  | Incongruent | 60 | 494.40 | 278.09 | 35.90 | 71.84 |
| Dayang  Dayang | Congruent | 68 | 362.94 | 148.33 | 17.99 | 35.90 |
|  | Incongruent | 66 | 354.98 | 154.00 | 18.96 | 37.86 |
| Kawan  Kawan | Congruent | 56 | 543.75 | 314.29 | 42.00 | 84.17 |
|  | Incongruent | 49 | 550.29 | 228.88 | 32.70 | 65.74 |
| Baju  Baju | Congruent | 62 | 445.84 | 275.60 | 35.00 | 69.99 |
|  | Incongruent | 49 | 467.39 | 305.39 | 43.63 | 87.72 |

** 95% Confidence Interval*


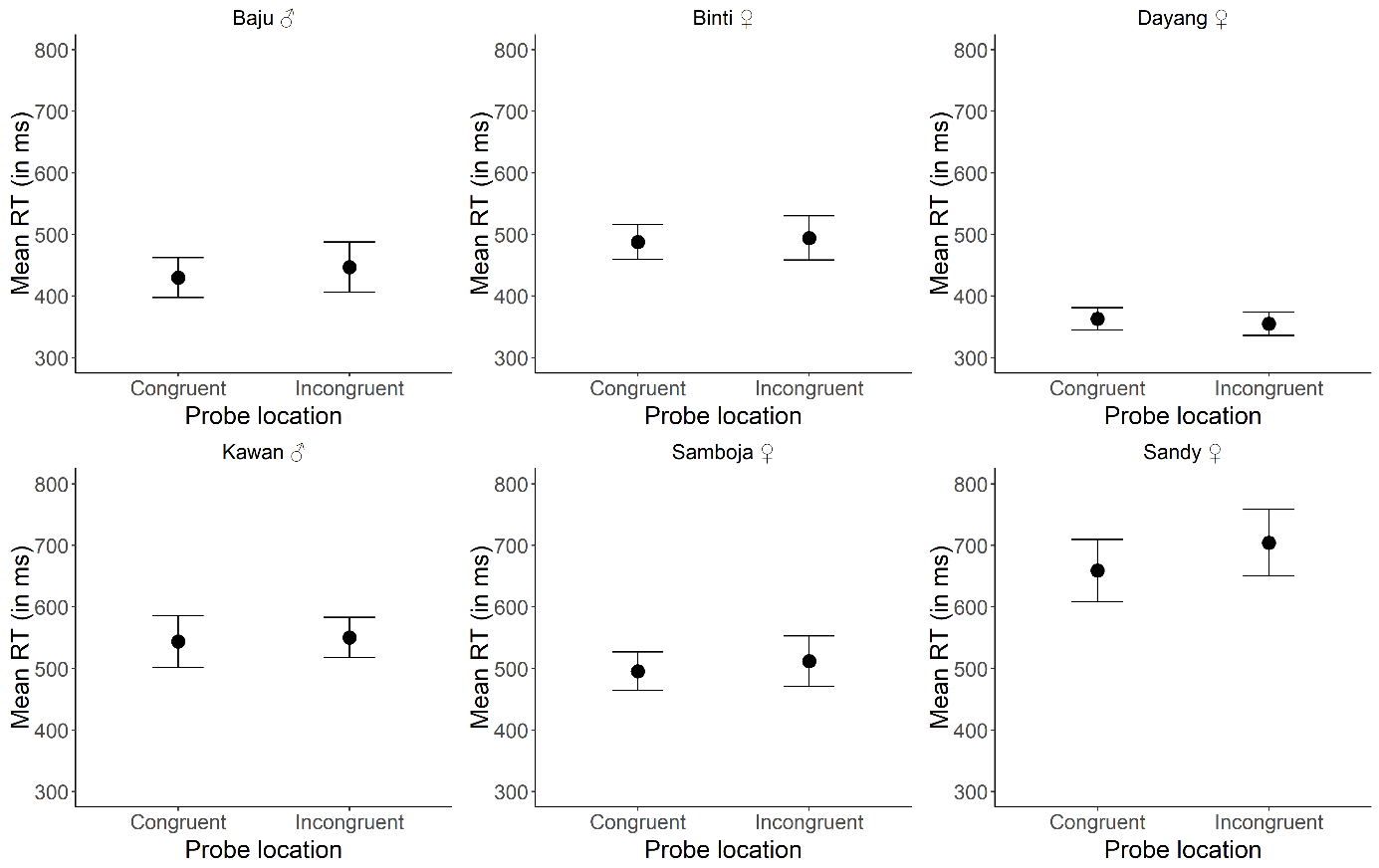


*Figure ESM3.* Mean reaction times per subject and per probe location. Error bars represent the SEM.

*Stimulus intensity*

We calculated a difference score by subtracting the intensity score of the distractor (non-probe) stimulus from the intensity score of the target (probe). The resulting *Difference Intensity Score* (DIS) ranged from -3.2 to 3.4, with a positive number indicating a higher intensity for the probe stimulus. We ran a Bayesian mixed (Student­­-*t*) model with reaction time as a continuous dependent variable and *DIS* as a continuous independent variable. We included nested random intercepts (sessions nested in subjects), a weakly informative Gaussian prior for the intercept (*M* = 500, *SD =* 100), a more conservative Gaussian prior for the intercept (*M =* 0, *SD =* 5), and default half Student-*t* priors with 3 degrees of freedom for the random effects and residual standard deviation. The model included the default 4000 iterations with 2000 warmups. We found no robust effect for the intensity difference score on reaction time (Table ESM5).

Table ESM5: Model output for Stimulus Intensity

| Parameter | Median Estimate | SD | 89% CI (lower) | 89% CI (upper) |
| --- | --- | --- | --- | --- |
| Intercept | 489.12 | 43.09 | 421.90 | 556.52 |
| Difference Intensity Score | -1.22 | 3.03 | -6.14 | 3.61 |
| Random effects |  |  |  |  |
| SD (Subject) | 109.96 | 43.37 | 59.37 | 188.18 |
| SD (Subject:Session) | 59.39 | 11.70 | 41.36 | 78.47 |
| *N*_obs_ = 798 |  |  |  |  |
| *N*_subj_ = 6 |  |  |  |  |

Note. *Difference Intensity Score* is a continuous variable

*Presence/absence of infants*

We created a new factor with four levels: infant present on both stimuli, infant absent on both stimuli, infant present on target (probe), infant present on distractor (non-probe). We ran a Bayesian mixed (Student­­-*t*) model with reaction time as a continuous dependent variable and *Infant Presence* as a categorical independent variable. We included nested random intercepts (sessions nested in subjects), a weakly informative Gaussian prior for the intercept (*M* = 500, *SD =* 100), a more conservative Gaussian prior for the intercept (*M =* 0, *SD =* 5), and default half Student-*t* priors with 3 degrees of freedom for the random effects and residual standard deviation. The model included the default 4000 iterations with 2000 warmups. We found no robust effect of the presence or absence of infants on reaction time (Table ESM6, Figure ESM4).

Table ESM6: Model output for *Infant presence*

| Parameter | Median Estimate | SD | 89% CI (lower) | 89% CI (upper) |
| --- | --- | --- | --- | --- |
| Intercept | 490.02 | 42.32 | 424.22 | 556.40 |
| Infant absent on both | 0.45 | 4.73 | -7.15 | 8.07 |
| Infant present on distractor | -1.67 | 4.73 | -9.18 | 5.82 |
| Infant present on target | -0.15 | 4.93 | -8.16 | 7.85 |
| Random effects |  |  |  |  |
| SD (Subject) | 108.33 | 42.98 | 59.83 | 185.48 |
| SD (Subject:Session) | 59.77 | 11.70 | 42.02 | 78.96 |
| *N*_obs_ = 798 |  |  |  |  |
| *N*_subj_ = 6 |  |  |  |  |

Note. *Infant presence* is sum-coded


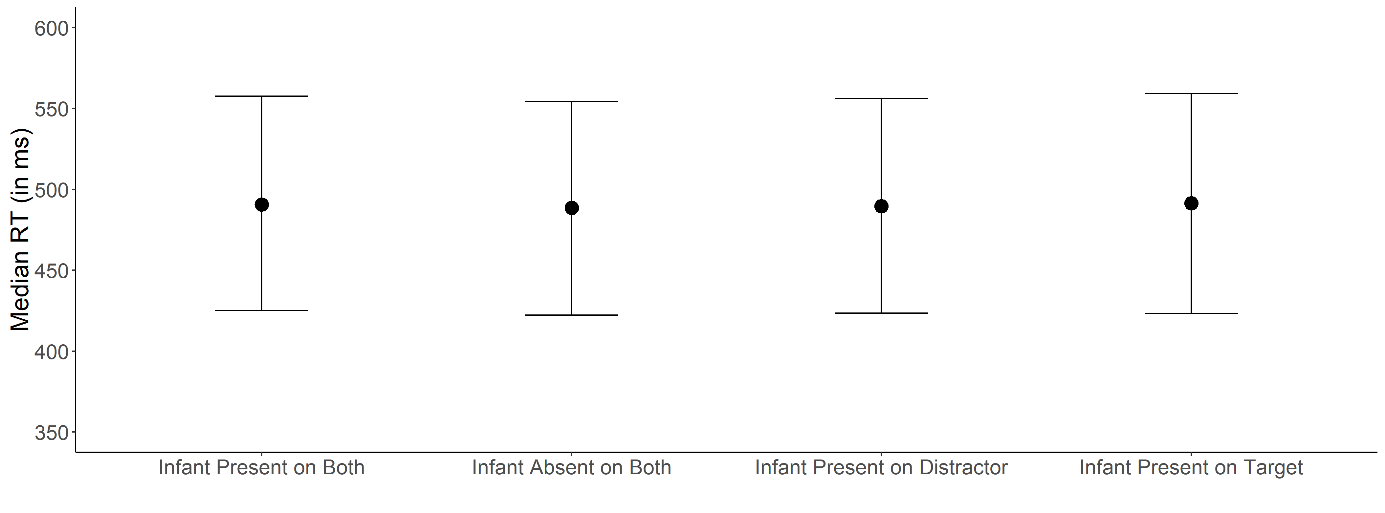


Figure ESM4. Median reaction times (in milliseconds) based on the presence or absence of infants on the target and/or distractor. Error bars represent the 89% credible interval.

As infants were mostly either present on both stimuli, or on neither, we created a subset containing only those trials and tested for an effect of infant presence on reaction time, and ran a Bayesian mixed model with the prior parameters included in the previous model. Again, we found no robust effect of the presence or absence of infants on reaction time (Table ESM7).

Table ESM7: Model output for *Infant presence (on both stimuli or neither)*

| Parameter | Median Estimate | SD | 89% CI (lower) | 89% CI (upper) |
| --- | --- | --- | --- | --- |
| Intercept | 490.02 | 42.73 | 424.16 | 557.08 |
| Infant absent on both | 1.51 | 4.25 | -5.37 | 8.31 |
| Random effects |  |  |  |  |
| SD (Subject) | 108.35 | 43.32 | 58.84 | 186.71 |
| SD (Subject:Session) | 57.65 | 12.22 | 38.92 | 77.29 |
| *N*_obs_ = 753 |  |  |  |  |
| *N*_subj_ = 6 |  |  |  |  |

Note. *Infant presence* is sum-coded

*Presence/absence of flanged males*

We created a new factor with four levels: flanged male present on both stimuli, flanged male absent on both stimuli, flanged male present on target (probe), flanged male present on distractor (non-probe). Using the same prior parameters as the Infant Presence model, we investigated the effect of *Flanged Male Presence* (categorical variable) on reaction time (continuous variable) in a Bayesian mixed model. We found no robust effect of the presence or absence of flanged males on reaction time (Table ESM8, Figure ESM5).

Table ESM8: Model output for *Flanged male presence*

| Parameter | Median Estimate | SD | 89% CI (lower) | 89% CI (upper) |
| --- | --- | --- | --- | --- |
| Intercept | 489.26 | 42.44 | 421.93 | 554.74 |
| Flanged male absent on both | 0.90 | 4.79 | -6.66 | 8.54 |
| Flanged male on distractor | 0.16 | 4.75 | -7.36 | 7.78 |
| Flanged male on target | -0.24 | 4.96 | -8.22 | 7.63 |
| Random effects |  |  |  |  |
| SD (Subject) | 110.34 | 44.42 | 59.86 | 188.28 |
| SD (Subject:Session) | 59.27 | 11.58 | 41.17 | 78.47 |
| *N*_obs_ = 797 |  |  |  |  |
| *N*_subj_ = 6 |  |  |  |  |

Note. *Flanged male presence* is sum-coded


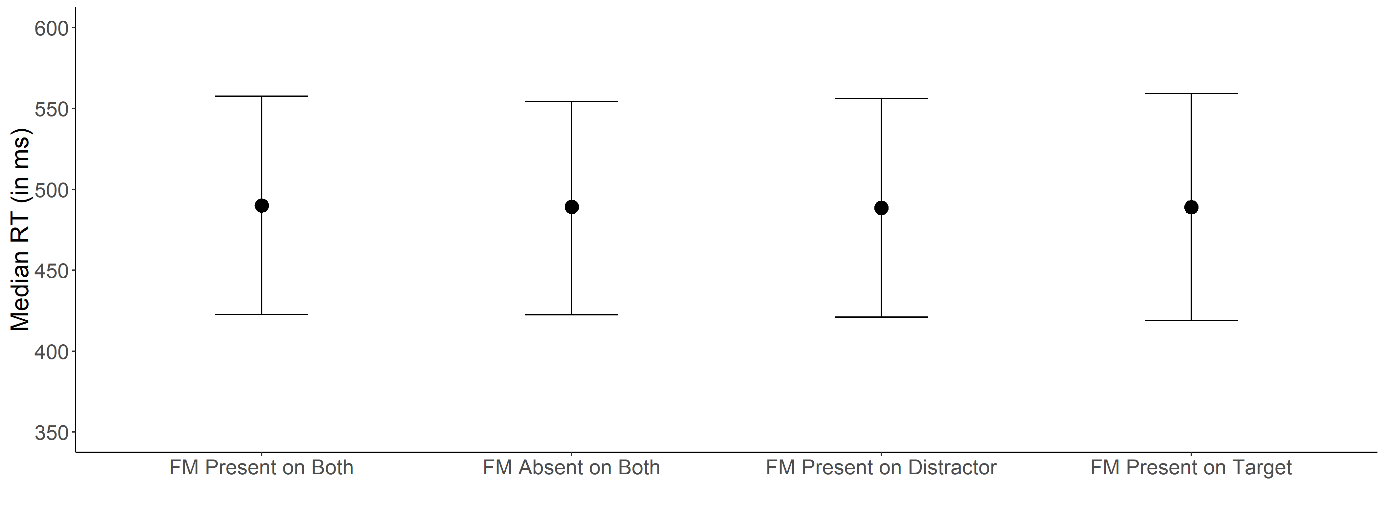


Figure ESM5. Median reaction times (in milliseconds) based on the presence or absence of flanged males (FM) on the target and/or distractor. Error bars represent the 89% credible interval.

As flanged males were mostly either present on both stimuli, or on neither, we created a subset containing only those trials and tested for an effect of flanged males presence on reaction time. Again, using the same prior parameters as the previous model, we found no robust effect of the presence or absence of flanged males on reaction time (Table ESM9).

Table ESM9­­­­: Model output for *Flanged male presence (on both stimuli or neither)*

| Parameter | Median Estimate | SD | 89% CI (lower) | 89% CI (upper) |
| --- | --- | --- | --- | --- |
| Intercept | 490.88 | 43.34 | 422.75 | 560.63 |
| Flanged male absent on both | 0.97 | 4.32 | -5.93 | 8.00 |
| Random effects |  |  |  |  |
| SD (Subject) | 111.09 | 43.51 | 60.82 | 191.25 |
| SD (Subject:Session) | 53.95 | 12.16 | 34.95 | 73.53 |
| *N*_obs_ = 752 |  |  |  |  |
| *N*_subj_ = 6 |  |  |  |  |

Note. *Flanged male presence* is sum-coded
